# Supplementary material for: Spatial and temporal patterns of public transit aerobiomes
Source: Microbiome. 2026 Jan 19;14:64. doi: 10.1186/s40168-025-02303-7 (PMC12896060; doi:10.1186/s40168-025-02303-7)
Supplement: Supplementary file 2 — Supplementary Material 1. [file 40168_2025_2303_MOESM1_ESM.docx]

**Supplementary Figures**

***
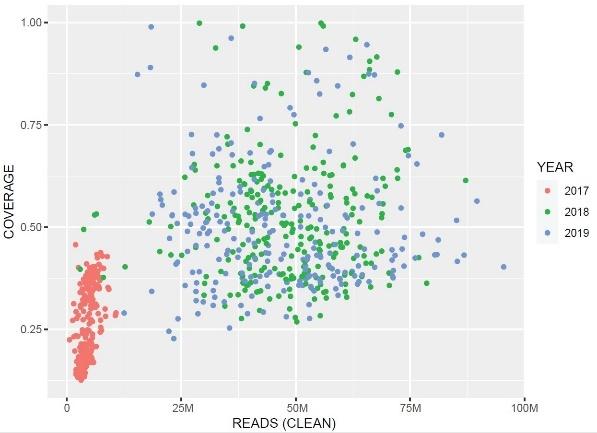
***

**Supplementary Figure 1.** **Nonpareil estimates of metagenomic coverage and sequenced diversity:** post trimming and removal of *Homo sapiens* and phi X 174. Red dots depict air samples from 2017, green from 2018, and blue from 2019. Read number, in millions, is shown on the x-axis, with coverage on the y-axis.


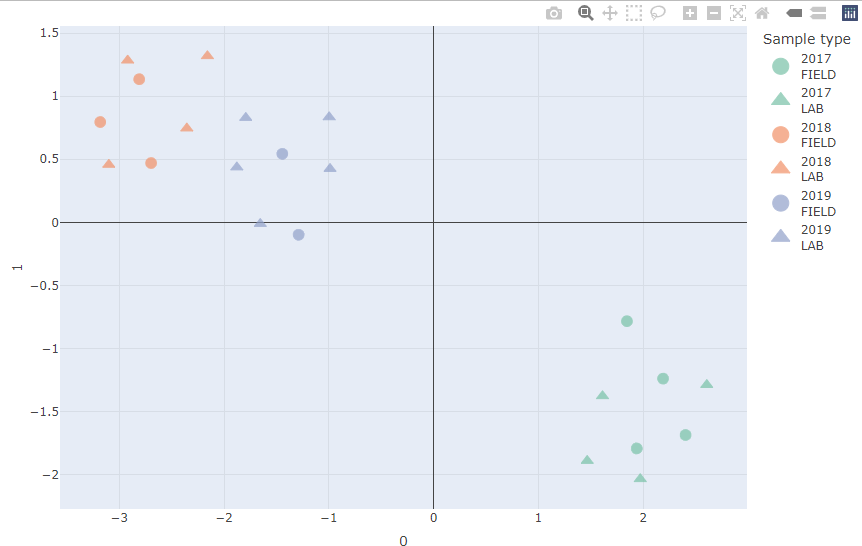


**Supplementary Figure 2. Beta diversity of negative control samples**: Difference test with UMAP between field- and lab-negatives post removal of *Homo sapiens* and Phix reads and separated to year.


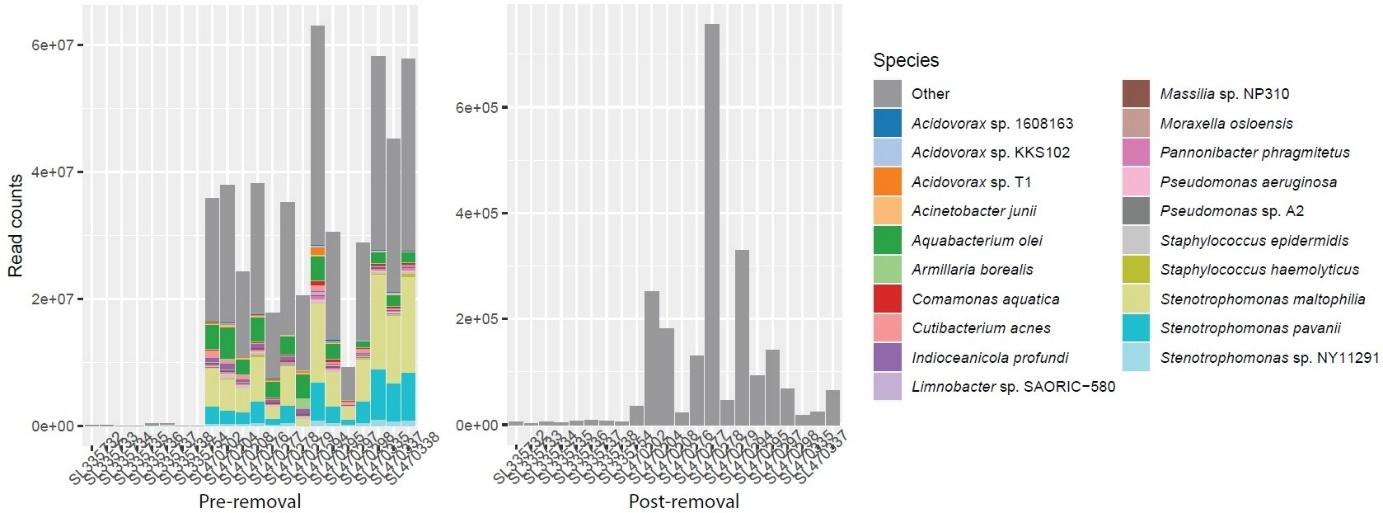


**Supplementary Figure 3 Relative read abundance of negative control samples pre- and post-contaminant removal**. The top 20 microbiome contaminants annotated with colour for all negative samples pre- (left) and post-removal of contaminating species taxon IDs.


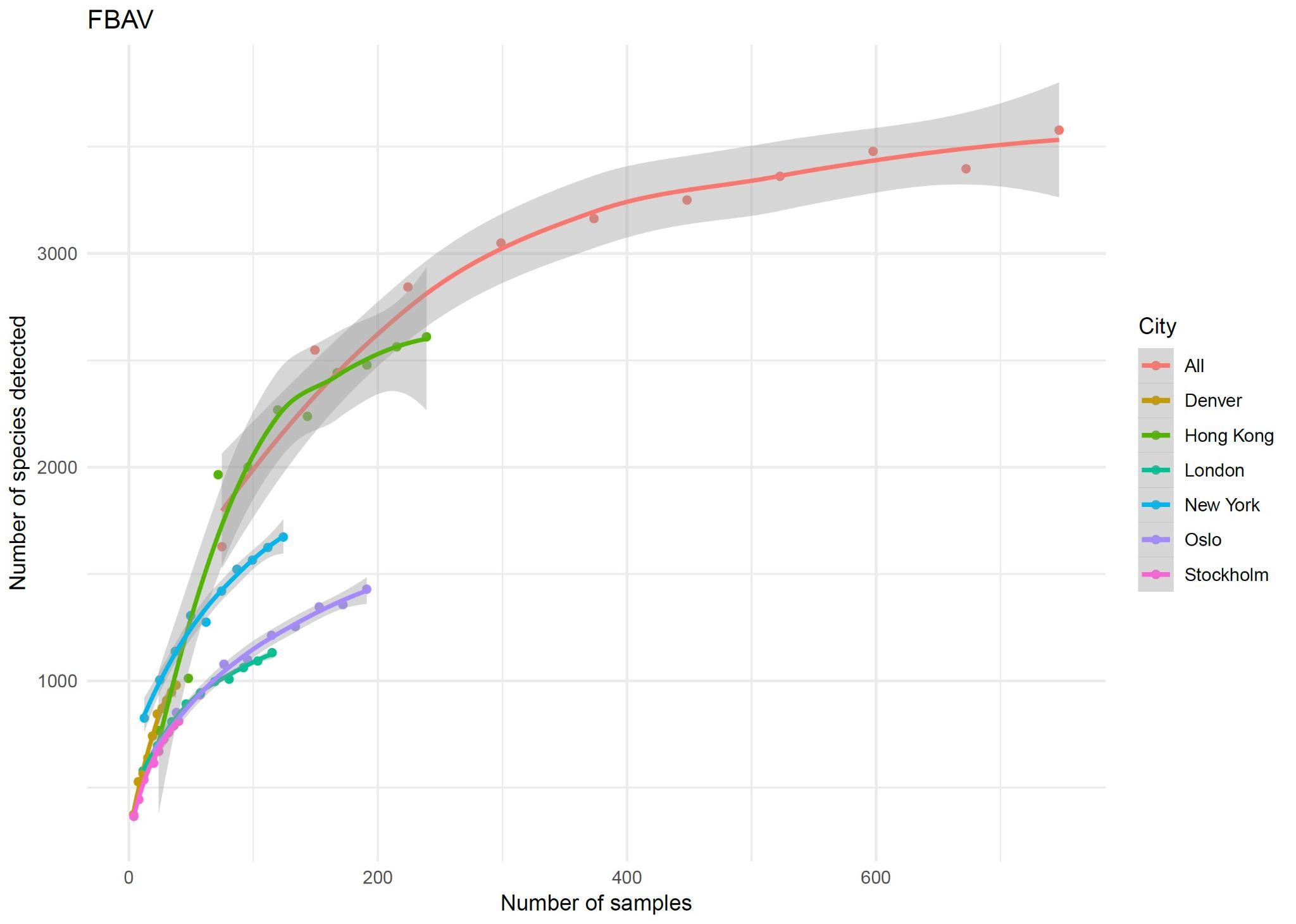


**Supplementary Figure 4. Rarefaction analysis of total sampled species diversity:** For all cities combined and separately against the FBAV database, as assessed by subsampling each dataset, 1,000 permutations

**Supplementary Figures 5-7. Interactive HTML beta-diversity plots:** with UMAP, for the microbiome (Fig. S5), bacteria (Fig. S6) and fungi (Fig. S7) separated by city and year. Taken from Fig. 3


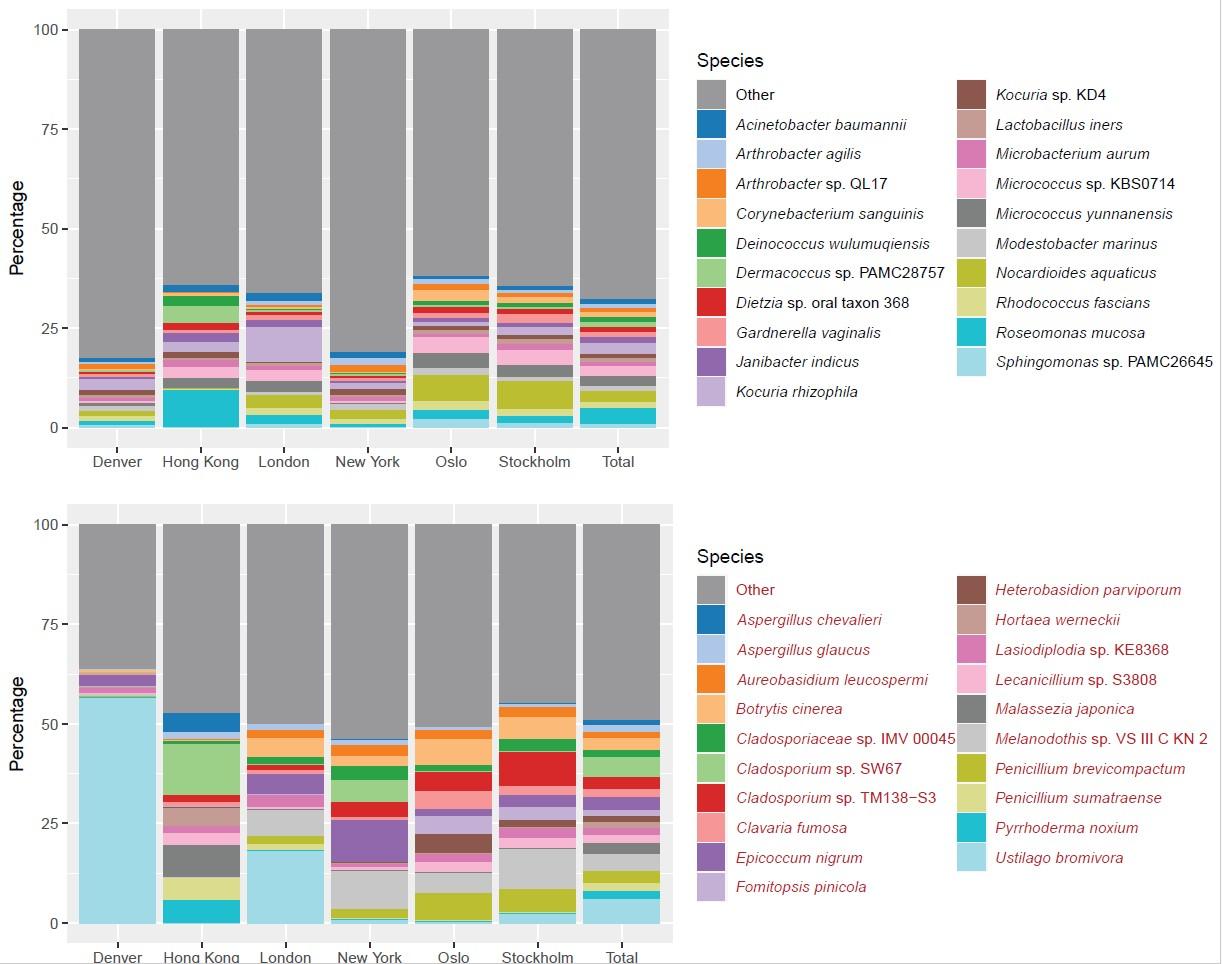


**Supplementary Figure 8. Relative classified species read abundance:** To the bacterial (top) and fungal (bottom) microbiome for all sampling years combined, classified to the FBAV database. The chart shows the top 20 most prevalent species in addition to all “other” species. Charts are divided into cities and the total of all six cities combined.
